# The price of blood is measured in iron

**DOI:** 10.1016/S0140-6736(17)32156-6

**Published:** 2017-09-21

**Authors:** Alan E Mast, Edward L Murphy

**Affiliations:** Blood Research Institute, BloodCenter of Wisconsin, Milwaukee, WI, USA (AEM); Department of Pathology and Department of Cell Biology, Neurobiology and Anatomy, Medical College of Wisconsin, Milwaukee, WI, USA (AEM); Department of Laboratory Medicine and Department of Epidemiology/Biostatistics, University of California, San Francisco, San Francisco, CA 94143, USA (ELM); and Blood Systems Research Institute, San Francisco, CA, USA (ELM)

Volunteer blood donors give about 500 mL of whole blood, approximately 10% of their total blood volume. After removal of plasma during processing, each mL of packed red blood cells contains 1 mg of iron. Thus, 200–250 mg iron are removed from the donor at each donation depending on their haematocrit. Since average iron stores are only 250 mg in women and 1000 mg in men, repeated donation produces iron deficiency in many donors.^1^ Iron deficiency induced by blood donation has potential for untoward effects, including impaired neurocognitive development in teenagers or in the fetus of a donor who becomes pregnant.^2^ Additionally, physicians might initiate unnecessary evaluations for gastrointestinal bleeding in male donors with iron deficiency.

There are two ways in which a blood donor can mitigate development of iron deficiency: take iron pills or lengthen their inter-donation interval. Although full recovery of iron stores following donation takes over 6 months for the average donor and over 90 days for an average donor taking a daily iron supplement,^3,4^ minimum inter-donation intervals range from 8 weeks to 16 weeks in different countries.

In this context, the findings of the INTERVAL study^5^ presented in *The Lancet* are of great importance. The investigators recruited 45 263 whole-blood donors between 2012 and 2014 from 25 centres across England and randomly assigned 22 466 men to 12-week (UK standard) versus 10-week versus 8-week inter-donation intervals, and 22 797 women to 16-week (UK standard) versus 14-week versus 12-week intervals. The primary outcome of blood units collected was increased with shorter compared with longer inter-donation intervals, as might be expected. Perhaps more important were the secondary safety outcomes. In men, at 2 years, mean haemoglobin was 143·1 g/L in the shorter inter-donation interval group versus 146·4 g/L in the longer inter-donation interval group (p<0·0001) and mean ferritin was 25·7 µg/L versus 36·3 µg/L (p<0·0001). Although overall quality of life, cognitive function, and physical activity did not appear to be adversely affected, participants in the shorter inter-donation interval group reported more symptoms possibly related to iron deficiency, including feeling faint, tiredness, breathlessness, dizziness, and restless legs (all p<0.0001 for men).

Strengths of this study include its randomised trial design, large study population, multicentre scope, and close adherence to the intervention by most participants. Additionally, the study included measurement of both biochemical (haemoglobin and ferritin) and subjective (symptomatology and quality of life) secondary safety outcomes. However, INTERVAL did have the weakness of potentially reduced generalisability inherent to many randomised trials. The proportion of donors participating among those approached was less than 50%, and enrolled donors lived closer to donation centres and had higher previous donation frequency than all UK donors. The findings are therefore strictly applicable to the UK and extrapolation to other countries should be done with caution.

What implications can be drawn from the study? First, over a 2-year period many donors can increase their donation frequency without a measurable effect on overall quality of life. As the authors conclude, the study suggests that for short-term periods blood collection agencies can safely use shorter donation intervals (8 weeks in men or 12 weeks in women) to meet shortages in periods of high demand. However, increased donation frequency comes with the cost of iron deficiency and related anaemia: about 25% of men and women at the most frequent inter-donation interval had iron deficiency and a third had at least one deferral for low haemoglobin.

Second, when blood supply is adequate or in surplus—as is the case currently in the USA^6^—longer intervals or iron supplementation should be used to prevent iron deficiency and associated symptoms.^7^ Some blood centres have already introduced ferritin screening and lengthened the inter-donation interval for donors found to have low ferritin concentrations.^8^ Given the advances in automated laboratory testing, information technology, and the high compliance of blood donors, individualised approaches for prevention of iron deficiency could be feasible, as has been done in Denmark.^9^ Teenage blood donors might be particularly susceptible to the negative consequences of iron deficiency and should be treated with increased care.^10,11^

The authors are to be commended for this groundbreaking study. Blood donors already provide the life-saving resource of blood through their altruistic donations and should not be asked to pay the additional price of iron deficiency. Blood centres now have the necessary tools to monitor their donors and adjust inter-donation intervals or provide iron supplementation.
